# Draft Genome Sequence of Salirhabdus euzebyi Strain Q1438

**DOI:** 10.1128/MRA.00246-20

**Published:** 2020-04-30

**Authors:** Rita Zgheib, Hussein Anani, Didier Raoult, Pierre-Edouard Fournier

**Affiliations:** a Aix Marseille Université, Institut de Recherche pour le Développement (IRD), Service de Santé des Armées, AP-HM, UMR Vecteurs Infections Tropicales et Méditerranéennes (VITROME), Institut Hospitalo-Universitaire Méditerranée Infection, Marseille, France; b Institut Hospitalo-Universitaire Méditerranée Infection, Marseille, France; c Aix-Marseille Université, Institut de Recherche pour le Développement (IRD), UMR Microbes Evolution Phylogeny and Infections (MEPHI), Institut Hospitalo-Universitaire Méditerranée-Infection, Marseille, France; d Special Infectious Agents Unit, King Fahd Medical Research Center, King Abdulaziz University, Jeddah, Saudi Arabia; Georgia Institute of Technology

## Abstract

In 2007, Salirhabdus euzebyi was first described as a bacterial isolate from a sea salt evaporation pond. As no genome sequence was previously available for this species, we performed whole-genome sequencing. The chromosome of strain Q1438 was 3,784,443 bp long with 36% G+C content, 3,830 protein-coding genes, and 74 RNA genes.

## ANNOUNCEMENT

The genus *Salirhabdus* was proposed in 2007 by Albuquerque et al. after the isolation of strain CVS-14 (=DSM 19612), the type strain of the type species *Salirhabdus euzebyi*, from a sea salt evaporation pond (1). We acquired *S. euzebyi* strain DSMZ 19612^T^ from the Deutsche Sammlung von Mikroorganismen und Zellkulturen GmbH (DSMZ). The strain was then deposited in the Collection de Souches de l’Unité des Rickettsies (CSUR) culture collection under the number Q1438. *S. euzebyi* was acquired for use in another study (H. Anani, R. Zgheib, B. Senghor, A. Fontanini, D. Raoult, and P.-E. Fournier, unpublished data). However, as no genome was previously available for it, we performed whole-genome sequencing of this species. In our laboratory, *S. euzebyi* strain Q1438 was grown on 5% sheep blood-enriched Columbia agar (bioMérieux, Marcy l’Etoile, France) and incubated in an aerobic atmosphere at 37°C for 48 h.

The genomic DNA (gDNA) of strain Q1438 was extracted on the EZ1 BioRobot (Qiagen, Germany) using the EZ1 DNA tissue kit. Sequencing was performed on a MiSeq sequencer (Illumina, Inc., San Diego, CA, USA) with the paired-end strategy, and the project was barcoded in order to be mixed with 23 other genomic projects prepared with the Nextera XT DNA sample prep kit (Illumina). The paired-end library was prepared as previously described (2). Automated cluster generation and paired-end sequencing with dual index reads were performed in a single 39-h run in 2 × 250-bp format. Total information of 6.36 Gb was obtained from a 682,000/mm^2^ cluster density with a cluster passing quality control filters rate of 94.2%. Within this run, the index representation for strain Q1438 was determined to be 3.67%. The 13,069,588 paired-end reads were filtered according to the read qualities using FastQC version 0.11.8 (https://www.bioinformatics.babraham.ac.uk/projects/fastqc/) with default parameters. Finally, the obtained forward and reverse reads were assembled using SPAdes version 3.13.1, as previously described (3, 4). The resulting draft genome sequence has a length of 3,784,443 bp with a G+C content of 36%. It is composed of 38 contigs (*N*_50_, 236,370 bp; *L*_50_, 6) and 36 scaffolds (*N*_50_, 300,736 bp; *L*_50_, 6), with 20× coverage. Genome annotation was conducted using Prokka version 1.14.5 (5) with the following options: coding sequences were predicted using Prodigal version 2.6 (6), and then the predicted bacterial protein sequences were searched against the GenBank (https://www.ncbi.nlm.nih.gov/genbank/) database using BLASTp. The search for tRNAs and transfer-messenger RNAs (tmRNAs) was done with ARAGORN version 1.2 (7), whereas rRNAs were predicted with Barrnap version 0.4. This annotation predicted a total number of 3,914 genes, out of which 3,830 were protein-coding genes and 84 were RNA-coding genes (10 rRNAs and 74 tRNAs). We performed a BLAST search for the predicted 16S rRNA against the GenBank database. *S. euzebyi* strain Q1438 showed a 16S rRNA similarity of 99.93% with *S. euzebyi* strain CVS-14^T^ (DSM 19612^T^; GenBank accession number NR_042538.1). Sixty four percent of genes were assigned to clusters of orthologous groups. Using the ARG-ANNOT (8) and VFDB (9) databases, neither resistance nor virulence genes were found in the genome of *S. euzebyi* strain Q1438.

### Data availability.

The draft genome and read sequences of *S. euzebyi* strain Q1438 (BioProject number PRJEB36962 and BioSample number SAMEA6593274) have been deposited at EBI/GenBank under accession numbers CADDWK010000001 through CADDWK010000038 and ERR3958931, respectively.
